# Role of Extracellular Vesicles in Liver Diseases

**DOI:** 10.3390/life13051117

**Published:** 2023-04-30

**Authors:** Viola Tamasi, Krisztina Németh, Miklós Csala

**Affiliations:** 1 Department of Molecular Biology, Semmelweis University, 1094 Budapest, Hungary; csala.miklos@semmelweis.hu; 2 Department of Genetics, Cell- and Immunobiology, Semmelweis University, 1089 Budapest, Hungary; nemeth.krisztina1@med.semmelweis-univ.hu; 3 ELKH-SE Translational Extracellular Vesicle Research Group, 1085 Budapest, Hungary

**Keywords:** extracellular vesicles, NAFLD, AFLD, hepatocarcinoma, viral hepatitis, autoimmune hepatitis, drug-induced hepatitis, liver, hepatocytes

## Abstract

Extracellular vesicles (EVs) are cell-derived membrane structures that are formed by budding from the plasma membrane or originate from the endosomal system. These microparticles (100 nm–100 µm) or nanoparticles (>100 nm) can transport complex cargos to other cells and, thus, provide communication and intercellular regulation. Various cells, such as hepatocytes, liver sinusoidal endothelial cells (LSECs) or hepatic stellate cells (HSCs), secrete and take up EVs in the healthy liver, and the amount, size and content of these vesicles are markedly altered under pathophysiological conditions. A comprehensive knowledge of the modified EV-related processes is very important, as they are of great value as biomarkers or therapeutic targets. In this review, we summarize the latest knowledge on hepatic EVs and the role they play in the homeostatic processes in the healthy liver. In addition, we discuss the characteristic changes of EVs and their potential exacerbating or ameliorating effects in certain liver diseases, such as non-alcoholic fatty liver disease (NAFLD), alcoholic fatty liver disease (AFLD), drug induced liver injury (DILI), autoimmune hepatitis (AIH), hepatocarcinoma (HCC) and viral hepatitis.

## 1. Introduction

The prominent scientific investigation of extracellular vesicles (EVs) began in the 1980s when Harding and coworkers discovered transferrin-receptor-containing EVs formed during the maturation of reticulocytes [1]. In the same year, Pan and Johnstone provided the first images of EVs from the same type of cells, and Rose Johnstone named the spherical structures derived from multivesicular body (MVB) fusion “exosomes”. Later, more and more research groups studied these structures [2]. Crawford and coworkers observed small particles of various content in platelet-free plasma and called them microparticles. They also demonstrated that EVs contain proteins, lipids and ATP [3,4]. By now, scientific knowledge about EVs has become widespread and noticeable. The importance of these particles can be justified with many physiological functions. They play a role in intercellular communication (e.g., they transfer signal molecules such as cytokines, adhesion receptors, regulatory nucleic acids) and carry other biomolecules (e.g., metabolites, nutrients, cofactors) and, therefore, they are capable of changing the composition and function of recipient cells. They can serve as diagnostic markers since their amount and/or composition changes in many diseases. Moreover, EVs can be used as natural drug delivery systems with low cytotoxicity and immunogenicity [5].

Because of the size and heterogeneity of EVs, it is very hard to adapt different, conventional analytical tools to detect these vesicles. Proper investigation of EVs implies specific protocols and requires certain functional and physical analyses. These are summarized in a frequently updated guideline called Minimal Information for the Study of Extracellular Vesicles (MISEV). For instance, electron microscopy and nanoparticle tracking analysis (NTA) are recommended for physical characterization of the EVs, and size exclusion chromatography is preferred to enrichment ultracentrifugation. Functional studies are also strongly recommended for the characterization of protein content. Recently developed methods or tools, such as bead-based flow cytometry, microNMR, the Nano-Plasmonic Exosome (nPLEX) Sensor, the Integrated Magnetic-Electrochemical Exosome (iMEX) Sensor and many other nanotechnology-based techniques can also be used besides conventional methods of protein analysis [6,7].

**Figure 1:**
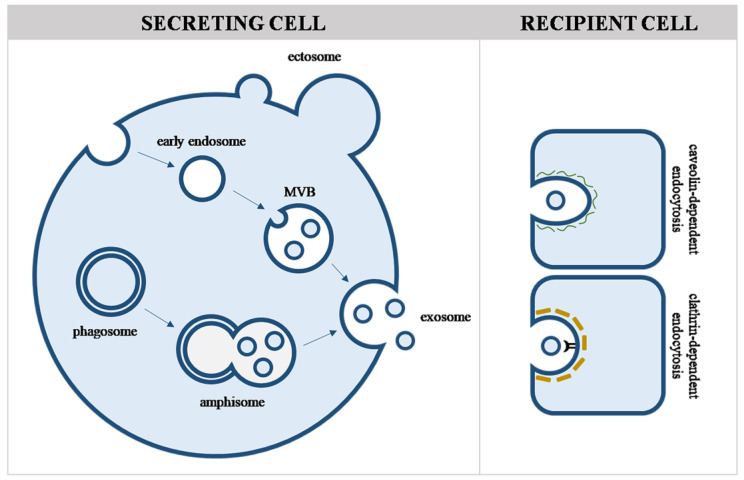
Formation of extracellular vesicles. Biogenesis of small EVs (exosomes) begins by maturation of early endosomes to MVBs or by the fusion of autophagosomes and MVBs to amphisomes. Small EVs generated with these mechanisms will be secreted by exocytosis. Ectosomes such as microvesicles are generated by plasma membrane budding and blebbing.

EVs can be broadly divided into two categories based on their biogenesis: exosomes and ectosomes [8]. Exosomes are small EVs of endosomal origin released by exocytosis of MVBs or amphisomes. The production of exosomes depends on the endosomal sorting complex required for transport (ESCRT) machinery. Alternative ways, such as transport through ceramide and sphingolipid membrane rafts, are also described [9,10]. By contrast, ectosomes are generated by plasma membrane budding and blebbing. Note that some ectosomes may also carry endosomal cargo components. Ectosomes include small-sized EVs (such as small ectosomes and arrestin-domain-containing protein 1-mediated microvesicles), medium-sized microvesicles and larger-sized apoptotic bodies (Figure 1).

All cell types in the body can produce and receive vesicles, but there are privileged tissues (e.g., the liver) for EV elimination. Some of the EVs taken up in these tissues may have regulatory function. On the other hand, EVs secreted from liver cells into the extracellular space may be received by neighboring cells or carried away by the blood to distant tissues. EV-based regulation may be paracrine, endocrine and possibly autocrine. Liver-derived EVs contain specific cargo, including certain molecules that are characteristic to liver cells [11].

The uptake mechanism by the recipient cell may be different, e.g., clathrin-dependent and caveolin-dependent endocytosis, macropinocytosis, phagocytosis and lipid raft-mediated uptake, with the first two being the most frequent. Since hepatocytes are polarized cells, EV uptake and release may be different on the apical (toward bile, apical EVs) and the basolateral (toward blood, basolateral EVs) sides. The mechanism of sorting EVs to different membrane sides in these cells is unknown. Recent studies suggest the role of the general guiding proteins, Rabs or the fusion/docking protein VAMP8 [12,13].

In this review, we discuss changes in the amount, composition, function, secretion, etc., of EVs in healthy and diseased livers.

## 2. EVs in Liver Physiology

### 2.1. EV Clearance by the Liver

Although the final destination of EVs is variable, based on pharmacokinetic studies, one can conclude that the liver precedes other organs in eliminating EVs independently of their protein content. These micro- or nanoparticles are removed mainly by Kupffer cells, but liver sinusoidal endothelial cells (LSECs) and hepatocytes are also involved in this process [14]. Kupffer cells, being liver macrophages, primarily clear those EVs that show some similarity to red blood cells or opsonized microorganisms. These have surface markers, such as CD44 or complement components CD55, CD59 or phosphatidylserine [15,16]. Nevertheless, regardless of their protein content, Kupffer cells eliminate all kinds of EVs. Other liver cells, such as hepatocytes and LSECs are also capable of taking up vesicles, but to a lesser extent [14]. Imai and coworkers showed that exosomes derived from the mouse melanoma cell line B16BL6 were taken up by macrophages in the liver and spleen, but not in the lungs [17]. Bala et al. showed that after intravenous administration of plasma containing miR-155-enriched exosome into miR-155-deficient mice, most of the miR-155 was found in the liver [18,19]. These exosomes were rapidly cleared from the serum, the maximum level was observed after 5 min and they were not detectable after 30 min. In another study, time-dependent changes were observed in different organs. The exosomes accumulated primarily in the liver, and microvesicles in the lungs. The hepatic level of exosomes was the highest in the first hour after administration, while the amount of microvesicles peaked in the first 60 min in the lungs and then in the liver [20]. EVs that escape from tissues such as the liver can reach their acceptor cells in other tissues or organs—even tumors—and be removed by those recipient cells [21].

The elimination of EVs shows high complexity; it depends on the donor and recipient cells and tissues, the size, content and surface proteins of EVs, and these differences impose a number of limitations on exosome-based drug delivery systems. However, the fact that EVs are predominantly cleared through the liver can be an advantage for vehicle-based therapies for liver diseases.

### 2.2. Liver as the Source of EVs

**Figure 2:**
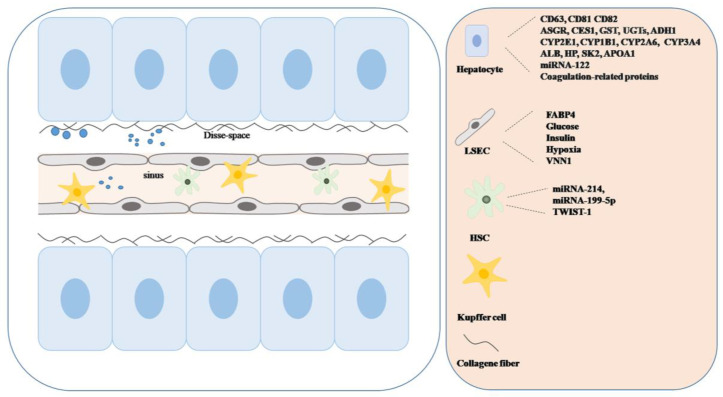
Content of EVs secreted by liver cells in the liver sinusoids. Liver cells (hepatocytes, Kupffer cells, liver sinusoidal endothelial cells [LSECs] and hepatic stellate cells [HSCs]) all secrete and receive EVs. The EVs differ in cargo, transmembrane and/or surface molecules. The figure represents major marker macromolecules in EVs originated from liver cells. Their cargo can be various: luminal, transmembrane or surface proteins; RNA, DNA or lipids.

Liver cells, such as hepatocytes, cholangiocytes, hepatic stellate cells (HSCs), liver sinusoidal endothelial cells (LSECs), Kupffer cells and various other immune cell populations, all secrete exosomes [22,23,24]. The amount and content of secreted EVs can change in response to diverse stimuli and under different disease conditions [25,26] (Figure 2).

#### 2.2.1. Hepatocytes

EVs released from hepatocytes express general EV markers, such as tetraspanin CD63, CD81 and CD82 or proteins involved in the endosomal pathway. They also contain hepatocyte-specific proteins, e.g., asialoglycoprotein receptor (ASGPR), cytosolic proteins including cytochromes, as well as secreted proteins such as coagulation-related proteins and apolipoproteins [27]. They carry drug-metabolizing enzymes (cytochrome P450 isoforms (CYPs), uridine diphosphate glucuronosyltransferases (UGTs), carboxylesterase-1 (CES1) and glutathione S-transferase (GST)) [22,27,28]. Remarkable amounts of CYP2E1, CYP1B1, CYP2A6 and CYP3A4 proteins and RNA suggest they may take part in detoxification processes occurring in blood plasma, and perhaps the potential role of plasma EV-CYPs or plasma-CYPs on pharmacokinetic changes should be taken into consideration during the drug development [29]. Moreover, these circulating liver-derived EVs can increase the metabolic capacity of recipient cells, and thus participate in detoxification or prodrug activation. These CYP-rich EVs may be considered as metabolic “sponges” that reach any tissues, cells or body liquids (e.g., liquor) where these drug metabolizing enzymes are physiologically absent [30]. Mobilization of metabolic processes by EVs can largely influence the pharmacokinetic properties of certain drugs.

Liver-derived exosomes carry tissue specific proteins/mRNAs/miRNAs, which can serve as potential biomarkers or drug targets in liver diseases. Some of these molecules are encapsulated, such as albumin (ALB), haptoglobin (HP), sphingosine-kinase 2 (SK2) and miRNA-122 [31], and others are expressed in the membrane, such as alcohol dehydrogenase-1 (ADH1) or apolipoprotein A-1 (APOA1) [32,33,34].

#### 2.2.2. LSECs

LSECs are specific endothelial cells that lack a basement membrane and are characterized by the presence of fenestrae. Many LSEC-derived exosomes contain sphingosine kinase 1 (SK1) that activates surrounding HSCs [35], thus promoting their transformation to myofibroblasts and potentiating their migration. EVs of HSCs are also known to be enriched in fatty acid-binding protein 4 (FABP4) that induces oncogenic processes once taken up by hepatocytes [36]. The hepatocyte–LSEC interaction is mutual. In vitro studies show that HepG2 cells secrete vannin 1 (VNN1) containing EVs after free-fatty acid treatment and these EVs can be taken up by endothelial cells. Since VNN1 plays a role in blood vessel formation and vascular diseases, this may be an EV-based link between liver and cardiovascular diseases [37].

#### 2.2.3. Kupffer Cells

Kupffer cells internalize EVs under physiological conditions. The captured EVs can be derived from various cells, or they can be even exogenous. However, the uptake is often selective and has a regulatory effect [38]. Altered EV uptake by Kupffer cells was observed under different pathological conditions, such as dyslipidemia [14].

#### 2.2.4. HSCs

These cells are responsible for fibrogenic processes and production of extracellular matrix proteins in the liver. They store vitamin A and retinol ester in their lipid droplets. They play a role in antigen presentation [39]. In quiescence, they secrete EVs containing miR-214, miR-199-5p and twist-related protein 1 (TWIST-1). These vesicles are taken up by neighboring quiescent HSCs (qHSC) and help keep them in quiescence by inhibiting connective tissue growth factor (CTGF) signaling. Upon activation of qHSCs, an increase in CTGF level upregulates the expression of profibrotic genes (e.g., collagen), and the activated HSCs (aHSC) activate additional neighboring HSCs in a paracrine manner [40].

#### 2.2.5. Cholangiocytes

These epithelial cells are responsible for bile production, participate in liver repair and liver injury [41]. Cholangiocytes are also exosome-releasing cells. They regulate and synchronize their own proliferation by using exosomes containing growth factors, extracellular signal-regulated kinase (ERK1/2) and miR-15a. These EVs interact with the cholangiocyte cilia and modulate cell division [42].

## 3. EVs in Liver Diseases

**Figure 3:**
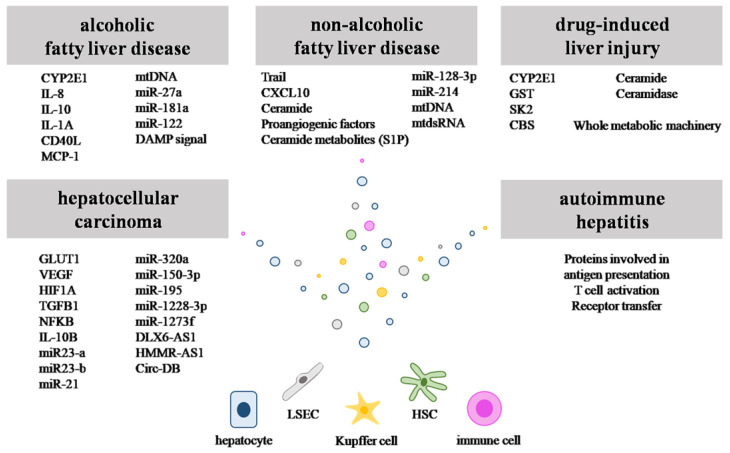
Content of EVs characteristic for various liver diseases. The schematic figure represents different cargos of EVs characteristic for liver diseases.

Chronic liver diseases affect a large percentage of the population worldwide, and there is growing evidence that Evs play an important role in liver pathophysiology. Multiple studies have been performed to explore the diagnostic and therapeutic potential of EVs in liver diseases. In the following section, recent knowledge about the importance of EVs in non-alcoholic fatty liver disease (NAFLD), alcoholic fatty liver disease (AFLD), drug induced liver injury (DILI), autoimmune hepatitis (AIH), hepatocarcinoma (HCC) and viral hepatitis will be discussed (Figure 3).

### 3.1. Role of EVs in NAFLD

NAFLD is a general term for many liver conditions, in which steatosis, ballooning and inflammation are present and are not caused by alcohol consumption [43]. Its prevalence is growing due to Western lifestyle, which is associated with obesity, elevated serum TG levels, impaired fasting glucose and excessive local hepatic ROS production [44]. Genetic factors, such as mutations in genes encoding patatin-like phospholipase domain–containing protein 3 (PNPLA3, I148M, rs738409), glucokinase regulatory protein (GCKR, rs780094) and squalene-synthase (FDFT1, rs2645424), also increase the risk of NAFLD [45]. The liver damage alters EV communication between the liver cells. The first findings in this area were obtained in animals fed a high-fat diet. They showed remarkable changes in the level and composition of circulating EVs compared those fed a control diet. The increase in the amount of circulating EVs correlated with the severity of steatohepatitis in mice [46]. In vitro studies demonstrated a significant increase in exosome production in hepatocytes treated with palmitic acid or cholesterol [47]. These lipids activate apoptotic pathways in the hepatocytes, stimulate immune response by macrophages and fibrogenesis and also induce hepatocyte-EV secretion through death receptor 5 (Trail receptor 2, TRAILR2). The latter is beneficial to hepatocytes as it reduces lipid accumulation in these cells [48]. Special hepatocyte-EVs contribute to macrophage activation. They contain TNF-related apoptosis-inducing ligand (TRAIL) and/or C-X-C motif ligand 10 (CXCL10) and turn macrophages into proinflammatory M1 type cells in the liver [48,49]. Moreover, the membrane of hepatocytes is enriched in 16:0 ceramide and ceramide metabolites, such as spingosine-1-phosphate (S1P). These lipids induce macrophage chemotaxis through an S1P1 receptor-mediated pathway. Prolonged activation of inositol requiring enzyme 1α (IRE1α) in lipotoxic endoplasmic reticulum stress enhances EV release. The massive secretion of EVs recruits macrophages to the site of inflammation [50]. Moreover, EVs encapsulate mitochondrial DNA (mtDNA) that also activates these immune cells through toll-like receptor 9 (TLR-9) [51]. Steatotic hepatocytes secrete EVs enriched in proangiogenic factors that induce angiogenesis and migration of LSECs [52].

In the inflamed fatty liver tissue, besides hepatocytes, other liver cells such as HSCs are also involved in the coordination of pathological processes. The EVs derived from hepatocytes under lipotoxicity contain miRNAs (e.g., miR-128-3p) that downregulate peroxisome proliferator-activated receptor gamma (PPAR-γ) expression in qHSCs. This leads to the upregulation of genes that are responsible for fibrogenesis, such as collagen-I (*Col-1*), α-smooth muscle actin (*Acta2*) and the tissue inhibitor of metalloproteinases-2 (*Mmp-2*) as well as genes that take part in proliferation, chemotaxis or wound-healing responses. The signal spreads between the cells by an as yet unknown mechanism, and one HSC activates many other HSCs [52]. Moreover, lipotoxic hepatocytes secrete EVs that contain mitochondrial double-stranded RNA (mtdsRNA) and, thus, activate toll-like receptor 3 (TLR-3) in qHSCs. The HSCs activated in this way stimulate cytokine production and release in Kupffer cells [53,54], the mechanism of which also remains to be elucidated. Activated HSCs also secrete miR-214-enriched vesicles. When these EVs are taken up by hepatocytes, their CTGF expression gets downregulated [55]. At the same time, an unknown mechanism triggers activated HSCs to secrete collagen, other extracellular proteins and profibrotic signals, such as tumor growth factor β (TGF-β), leptin (LEP) and platelet-derived growth factor (PDGF). These proteins induce the remodeling of hepatocytes, reduce hepatic elasticity and elevate portal blood pressure [56].

Extrahepatic EVs, such as EVs from adipose tissue, also contribute to NAFLD, mostly by the modulation of extracellular matrix proteins and promotion of fibrosis. They alter the expression of many genes, such as metalloproteinase-1/7 (*MMP-1/7*), integrin α*ν*β-5 (*ITGA5*) and plasminogen activator inhibitor-1 (*SERPINE1*), in hepatocytes or metalloproteinase-1/4/9 (*MMP-1/4/9*), ITGA5, integrin α*ν*β-8 ((*ITGA8*) and mothers against decapentaplegic homolog 3 (*SMAD-3*) in HSCs [57].

### 3.2. Role of EVs in AFLD

AFLD is a term that incorporates different symptoms of the liver after alcohol overconsumption. Alcohol interferes with lipid metabolism and increases the deposition of triglycerides, phospholipids and cholesterol esters. During progression of AFLD there are stages, starting with fatty liver, alcoholic hepatitis and chronic hepatitis with liver fibrosis and finally cirrhosis or liver cancer. On the cellular level, AFLD can be characterized with hepatocellular injury, ballooning and inflammation caused by innate immune cells. Neoantigens formed during alcohol metabolism triggers adaptive immune responses that are persistent and make the inflammation chronic and in parallel promote extracellular matrix deposition and fibrosis [58].

Over the last decade, there is increasing evidence that EVs play a role in the pathomechanism of AFLD. Since alcohol is metabolized in the liver, the first studies were focusing on P450 enzymes and their amount in hepatocyte-EVs. Cytochrome P450 Family 2 Subfamily E Member 1 (CYP2E1) is one of the enzymes that metabolizes alcohol to acetaldehyde in the liver; thus, it is highly responsible for liver toxicity. Rahman and coworkers discovered that EVs containing CYP2E1 also take part in alcohol metabolism [59]. Production of acetaldehyde either way can lead to toxicity and apoptosis of hepatocytes [31]. The steps of liver inflammation where EVs have importance are very similar to those found in NAFLD. For example, some EVs contain mtDNA [60] that recruits neutrophils, and others carry interleukin-8, interleukin-10 or interleukin-1a (IL-8, IL-10, IL-1a) [61].

Like in NAFLD, the activation of hepatocytes is followed by the mobilization of monocytes. Increased amounts of hepatic EVs in AFLD are taken up by monocytes and are responsible for their activation. The miRNAs, such as miRNA-122 and CD40L, turn these cells to M1-macrophages, which further increase infiltration of additional monocytes and Kupffer cells with chemoattractants such as monocyte chemoattractant protein 1 [62,63]. DAMP signals (danger-associated molecular pattern e.g., mtDNA) are also present in some of the EVs. These vesicles take part in the immune response by binding to toll-like receptor-9 and T cell priming [64].

In AFLD, HSCs can be activated with EVs originated from hepatocytes. These EVs carry miRNA-27a and miRNA-181a that activates qHSCs and in the long run and induces fibrogenesis [65].

### 3.3. EVs and Autoimmune Hepatitis

Autoimmune liver disease (AILD) is the common name for a variety of clinical conditions of the liver that are associated with autoimmune injury and inflammation [66]. They are characterized by elevated circulating immunoglobulin levels and liver enzymes as well as lymphocyte infiltration and autoantibody production. Due to the immune-mediated origin of AILD, certain genetic variants of the human major histocompatibility or human leukocyte antigen (MHC/HLA) complex are among the most significant risk factors of the disease. In the later stages of AILD, hepatic fibrosis and cirrhosis and even hepatocellular carcinoma can develop [67].

Antigen presentation plays a central role in autoimmunity, i.e., the production of antibodies against self-antigens. A small portion of the antigen (called an epitope) is presented on MHC-I or MHC-II surface proteins (major histocompatibility complex) [68]. The loaded MHC proteins are recognized by T cells. MHC-I is present on professional antigen-presenting cells (APCs), which activate CD8^+^ cells, and MHC-II proteins are found on all cells and prime CD4^+^ T cells. In the liver, the professional antigen-presenting immune cells include Kupffer cells, T cells and dendritic cells, while non-professional ones include hepatocytes, LSECs and HSCs [69]. The resulting immune response to an antigen is a complex process, which depends on the coordinated action of all primed cell types and their communication with each other via soluble molecules and/or EVs [70]. For instance, antigen-MHC-I complexes transferred by EVs from a professional APC to other cells (e.g., loaded MHC-I from Kupffer cells to HSCs) may turn the latter to professional APCs. Such an “infection” by loaded EVs may initiate a strong, CD8^+^ T cell-dependent immune response or induce apoptosis in the recipient cells. On the other hand, an EV-mediated transfer of loaded MHC-II proteins can enrich professional APCs with a complex that allows these cells to prime CD4^+^ T lymphocytes. Hepatic CD4^+^ T cell activation often results in the induction of Foxp3^+^ IL-10^+^ CD4^+^ T_reg_ cells rather than reactive T cells [71,72]. Some EVs maintain tolerance, and hence are called “tolerosomes” [73]. Interestingly, tolerosome production requires MHC-II activation on the small intestinal epithelial cells, which suggests the role of environmental antigens (e.g., from microbes or food) [74].

### 3.4. EVs and drug-Induced Liver Injury

Drug-induced liver injury (DILI) and herbal-induced liver injury (HILI) can present symptomatically as either acute or chronic liver diseases. Although DILI can be caused by various drugs, e.g., chlorpromazine, isoniazid [75], azathioprine or infliximab, the leading cause of this liver disease is acetaminophen/paracetamol (APAP) [76]. APAP is metabolized by CYP2E1 into a reactive electrophile N-acetyl-p-benzoquinone imine (NAPQI) [77], which can easily reach toxic concentrations. The harm caused by NAPQI is due to its reaction with sulfhydryl groups of proteins and glutathione (GSH) [78], and its redox cycle generating reactive oxygen species (ROS) which can lead to mitochondrial injury and cell death [79,80].

Many studies have shown that APAP administration increases the number of plasma EVs. Hepatocytes can receive CYP2E1 proteins and mRNAs by taking up these EVs [81,82], and the same EVs have been shown to carry the detoxifying glutathione S-transferase (GST) enzyme that conjugates NAPQI with glutathione [83]. The latter findings provide evidence that EVs may transfer whole metabolic machineries between stressed cells. The cargo may strengthen the recipient’s protection, but they are not always beneficial. Other biomolecules transferred by EVs in APAP toxicity include ceramides, ceramidase, sphingosine kinase 2 (SK2) and cystathionine-β-synthase (CBS) [84,85], and these EVs have been shown to decrease the level of the antioxidant ascorbic acid and arginine [85]. Moreover, APAP-EVs modulate the immune response in the liver by the induction of TNF-α and IL-1β [82].

There are other drugs and poisons that cause DILI, e.g., polyaromatic hydrocarbons present in the burned food or cigarette smoke (benzo[a]pyrene (BP), dibenzo[a,h]anthracene (DBA) and pyrene (PYR)). They also increase the amount of EVs [86] in the liver; however, they activate EV production in distinct ways. For example, BP- and DBA-induced EV production is regulated by the aryl hydrocarbon receptor (AhR), while PYR-induced EV production is regulated by another xenosensor, the constitutive androstane receptor (CAR). CYP induction is among the downstream effects of these xenosensors (CYP1A for AhR; CYP2B for CAR). These toxic molecules may also lead to apoptosis through increased caspase 3/7 (CAS3/7) activity [87]. These findings highlight that xenobiotics not only modulate the expression of drug metabolism-related genes but also differentially modulate various EV biogenesis pathways by influencing different molecular mechanisms.

### 3.5. EVs and Liver Cancer

Hepatocellular carcinoma (HCC) is the most common primary cancer of the liver, with a high recurrence rate and a low 5-year survival rate. Main risk factors include chronic hepatitis, fatty liver disease, diabetes, alcohol consumption and liver cirrhosis [88].

It is generally known that different tumor cells, such as HCC cells, secrete more vesicles than healthy cells. These tumor cell-derived EVs play a role in metastatic processes, multifocal growth, microenvironment changes and modulation of the immune response. Secreted HCC-derived EVs can affect other HCCs, healthy liver cells or other cells, including immune cells.

There are many, non-cancerous cell types in the tumor microenvironment (e.g., Th cells) that play an antitumorigenic role or to the contrary, promote tumorigenesis. These cells communicate with HCC cells through chemical signals and/or EVs.

#### 3.5.1. Regulation of HCC Tumorigenesis by Macrophage-Derived EVs

Tumor-associated macrophages (TAMs) are present in the microenvironment of HCCs, and they can be polarized to M1 or M2 cells [89]. These cells secrete various EVs to regulate cell proliferation, apoptosis and metastatic processes in HCC cells. For example, TAMs that have M1 phenotype secrete anti-tumorigenic EVs, which activate T cells and strengthen the immune response against cancer. They can be used as an effective adjuvant in cancer vaccines [90]. In contrast, M2 TAMs promote HCCs tumorigenesis by suppressing the immune response [89]. EVs secreted by these cells decelerate CD8^+^ T cell proliferation via miR-21-5p/YOD1/YAP/CTNNB1 axis (YOD1: Deubiquitinase YOD1, YAP: Yes1 Associated Transcriptional Regulator, CTNNB1: catenin β1), and in HCCs, decrease the amount of miR-125a/b (targeting CD90, a known promoter of proliferation) [91,92].

#### 3.5.2. Regulation of HCC Tumorigenesis by Adipocyte-Derived EVs

Adipocytes provide various molecules to cancer cells, and these molecules fuel a higher metabolic rate/energy production to drive proliferation and metastatic processes. Studies with mice on a high-fat diet indicated that adipocyte-derived EVs are enriched in miR-23a/b, vascular endothelial factor (VEGF), glucose transporter 1 (GLUT1) and hypoxia inducible factor 1 subunit alpha (HIF1α). It has been suggested that these adipocyte derived EVs might promote tumor growth through inducing cell proliferation by decreasing the level of the anti-tumor miRNA-34a and upregulating the pro-cancer ubiquitin-specific peptidase 7/Cyclin A2 signaling pathway (USP7/CCN2A) [93,94]. These two processes are orchestrated by vesicular circular RNAs (e.g., EV-cirk-DB [*EV*-*circ*-de-ubiquitination [95].

#### 3.5.3. Fibroblast-Derived EVs in HCC

Cancer-associated fibroblasts (CAFs) are activated by hepatocyte EVs that carry miR-21. The activation of CAFs correlates with various oncogenic parameters, such as the increase in β-catenin, mTOR or STAT3 levels [96]. Activated CAFs secrete vesicles enriched in miRs that may have pro- or antitumorigenic effects. Some CAF-EVs contain miR-320a (targeting PBX3 tumor growth promoter), miR-150-3p (inhibiting migration/invasion) and miR-195 (targeting VEGF and cell division cycle proteins, such as CDC42, CDK1, CDK4, CDK6 and CDC25) [97,98,99]. Other CAF-derived EVs transfer miR-1228-3p that downregulates placenta-associated 8 (PLAC8) oncogene and activates the phosphatidylinositol 3-kinase/protein kinase B (PI3K/AKT) pathway that increases chemoresistance of these cells [100].

#### 3.5.4. Mesenchymal Stem Cell (MSC)-Derived EVs and HCC

MSCs are present in many tissues, and their therapeutic value has been reported in many cancer types. MSC therapy has also been successfully applied in liver diseases as it alleviates liver damage, diminishes inflammation and promotes liver tissue regeneration [101,102,103,104]. It is believed that MSCs communicate with cancer cells through EVs. MSC-EVs are immunogenic vesicles containing CD107, CD63, CD81, CD29, CD73, CD90 and CD44, mRNAs, miRNAs (-132, -21, -21-5p), cytokines, chemokines, immunomodulators and growth factors [105,106,107,108]. MSC-EVs modulate the cells of both innate and adaptive immune systems, resulting in immunosuppression. Other MSC-EVs in the hepatic microenvironment have therapeutical potential, such as those delivering superoxide dismutase 2 (MnSOD) into hepatocytes and, thus, help suppress oxidative stress as it has been demonstrated in vitro [109] in a CCl_4_-induced liver tumor model [110]. Moreover, MSC-EVs have a significant effect on hepatic tumor growth by blocking the cell cycle and inducing apoptosis or necrosis [111].

The preconditioning of MSCs as part of an ex vivo therapy improves the function and therapeutic potential of MSCs. Preconditioning by IFN-γ resulted in the secretion of MSC-EVs that promoted a more efficient liver tissue repair and function [112].

#### 3.5.5. Cancer Stem Cell (CSC)-Derived EVs and HCC

CSCs are a subpopulation of stem-like cells within tumors that exhibit characteristics of both stem cells and cancer cells. They play important roles in tumor relapse and metastasis [113]. The EVs secreted from these cells participate in the regulation of metastatic processes, tumor growth, angiogenesis and maintaining the stem cell phenotype [114,115]. They have a specific role in tumor escape and modulation of immune responses in the tumor microenvironment. For example, CSC-EVs carry IL-10, TGFβ1 and NFκB that inhibit T-cell activation and activate anti-inflammatory processes. On the other hand, CSC-derived EVs increase the expression of MMP9 and VEGF and decrease the level of TIMP1 (metallopeptidase inhibitor 1) mRNA in the liver of mice. In addition, these vesicles inhibit apoptosis by inducing the BCL2 apoptosis regulator (BCL2) and decreasing the BCL2-associated X apoptosis regulator (BAX) and p53 protein expression [116,117]. Collectively, these findings suggest that CSC-derived EVs promote growth, survival and cell invasion in hepatocellular carcinoma.

#### 3.5.6. Hypoxia and HCC

Hypoxia promotes the production and release of EVs from cancer cells through a largely HIF-1α-dependent regulation [118]. HCC-derived EVs affect cell growth, invasion and metastasis in a paracrine manner, and the hypoxic HCC-derived EVs contain specific miRNAs, such as miRNA-1273f (targeting LHX6, homeobox protein). A decreasing level of the homeobox protein in the recipient cell inhibits the Wnt/β-catenin signaling pathway, thus delaying tumorigenesis [119]. Long non-coding RNAs, such as DLX6-AS1 or HMMR-AS1 found in hypoxic HCC-EVs, diminish pro-inflammatory processes (e.g., increase M2 macrophage polarization) in the tumor microenvironment. DLX6-AS1 induces M2 activation by competing with miR-155 to regulate C-X-C Motif Chemokine Ligand 17 (CXCL17). HMMR-AS1 activates M2 cells through an miR-147a/ARID3A pathway (AT-Rich Interaction Domain 3A) [120]. M2 cells make the tumor microenvironment less immunogenic, and thus promote tumorigenesis, invasion and migration of HCCs.

Tumor expansion requires new blood vessels to provide oxygen, glucose and other nutrients to the cells, and hypoxia is one of the key regulators of tumor angiogenesis. EVs have both pro- and anti-angiogenic properties. One of the mechanisms by which HCC-EVs affect angiogenic endothelial cells under hypoxia is the upregulation of miR-155 [121]. Human studies showed that this effect has a clinical relevance, since HCC patients with high miRNA-155 levels had shorter overall and disease-free survival [122]. Moreover, miRNA-155 expression significantly correlated with the expression of VEGF and HIF-1α and microvessel density [121]. There are other HCC-EV miRNAs that regulate angiogenesis, but they do not seem to depend on hypoxia [123,124,125]. EVs participate in the formation of hepatic metastasis of primary cancer types. For example, miR-135a-5p-enriched EVs from colorectal cancer that are taken up by Kupffer cells promote metastatic processes modulating the kinase 2-YAP-MMP7 axis [126]. Hypoxia may be a risk factor for other liver diseases beside cancer. In vitro studies of Hernández and coworkers showed that hypoxic conditions induce fibrosis in a fatty liver. EVs from hypoxic, fat-laden HepG2 cells initiate fibrotic processes in LX-2 cells (a human hepatic stellate cell line). These Evs transfer biomolecules that activate genes, such as TGF-β1, CTGF, collagen-I and α-SMA [127].

## 4. Viral Hepatitis

### 4.1. Viruses and Evs

Viruses (especially encapsulated RNA viruses) and Evs have more in common than it was previously thought. They are very similar in size, i.e., virion diameters range up to 1000 nm but can be as small as 30 nm (e.g., hepatitis A virus [HAV] and hepatitis C virus [HCV] have 30 nm and 50 nm diameters, respectively) [128]. Certain viruses can possess a membrane envelope enriched in glycosphingolipids and cholesterol assuming a structure very similar to that of Evs. Both of them contain nucleic acids. While viruses carry their genome (ds or ss DNA or RNA), Evs contain all kinds of nucleic acids (dsDNA, ssDNA, mRNA, miRNA, lncRNA, fragments, etc.) [129,130,131]. They also share the same biogenesis pathways, i.e., the endosomal pathway and ESCRT machinery or budding from the plasma membrane, and they leave the cell with exocytosis [132,133]. Evs, like some viruses, have glycan molecules on their surfaces. Viral glycans are derived from the host cells (e.g., blood antigen A), and viruses can use these molecules to bind to cells or evade the immune system [134,135,136,137]. Both EVs and viruses enter the recipient cells by endocytosis (e.g., using ICAM molecules on their surfaces) and release their content to the cytoplasm [138] (Table 1).

### 4.2. Types of Viral Hepatitis

**Figure 4:**
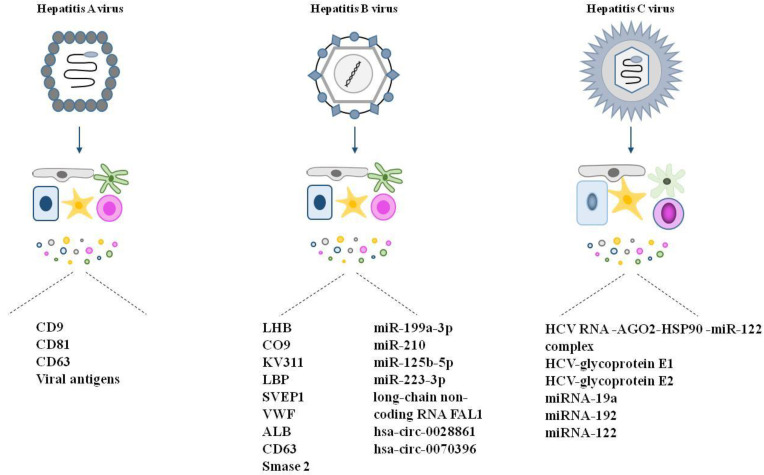
Content of liver cell-derived EVs after viral infections. The figure represents specific cargo of EVs secreted by cells infected with hepatitis A, B or C.

Viral hepatitis can be caused by five different viruses (hepatitis A, B, C, D and E viruses). The infection can be acute or chronic (for hepatitis B, C and D). EVs play an important role in these infections as they can either increase or decrease the risk and progression of the disease. For example, they can carry viruses between cells, involved in virus replication or shut down immune processes. On the other hand, immune cell-derived EVs can participate in the antiviral response and help to eliminate viruses [139] (Figure 4).

#### 4.2.1. Hepatitis A

Hepatitis A virus (HAV) is spread through the consumption of food or water contaminated with the feces of an infected person. Symptoms of this disease include fever, loss of appetite, diarrhea, nausea, dark-colored urine and jaundice (WHO, 2022) [140]. Infected liver cells spread two types of virions. i.e., non- and quasi-enveloped (eHAV). Non-enveloped virus particles are present in the feces, while eHAVs are present mostly in the cells and blood, and in the latter, the envelope protects the enclosed virus from immune cells. Growing evidence supports that eHAVs are EV-transmitted virions. McKnight and coworkers demonstrated that eHAVs have a density of ~1.05–1.10 g/cm^3^, which is similar to that of exosomes and that eHAV particle-enriched fractions contain the exosome markers CD9, CD81 and CD63 [141]. Moreover, eHAVs, similarly to EVs, use the ESCRT machinery (ALG-2-interacting *protein* X (Alix), vacuolar protein sorting 4 homolog A (VPS4A, VPS4B) for budding. HAVs packed in vesicles can be transmitted to other cells. The phenomenon that EV-encapsulation protects HAVs from being recognized by the immune cells/neutralizing antibodies is referred to as Trojan exosome, and it facilitates virus spreading in the liver [142]. Virus-loaded EVs bind to HAV cell receptor 1 (HAVCR1) and the NPC intracellular cholesterol transporter (NPC1) in the recipient cell [143]. This virus encapsulation can also increase the tropism of the virus. The great diversity of EV surface proteins allows eHAVs to spread not only in the liver but also in other organs [144].

#### 4.2.2. Hepatitis B

Hepatitis B is a potentially life-threatening liver infection caused by the hepatitis B virus (HBV). It spreads with blood, e.g., from mother to child, transfusion, liver transplantation, etc. It can cause chronic infection, putting affected people at high risk of death from cirrhosis and liver cancer (WHO, 2022) [145]. HBV is an enveloped virus that binds to sodium taurocholate cotransporting polypeptide (NTCP) of liver cells for entry. Wu and coworkers discovered that EVs secreted by HBV-infected cells contain virus particles, intact virions. They also demonstrated the presence of an HBV surface protein, i.e., Large Hepatitis B Virus Surface Antigen (LHB) on the surface of the vesicles. These new discoveries provide strong evidence for the role of EVs in virus replication and transmission [146]. It has been revealed in humans that EVs from HBV-infected cells contain different proteins compared to those secreted from healthy cells [147]. The diseased cells and their EVs have elevated amounts of complement component C9 (C9); immunoglobulin kappa variable 3-11 (KV311); lipopolysaccharide binding protein (LBP); immunoproteins [148,149,150], Sushi, Von Willebrand Factor Type A, EGF And Pentraxin Domain Containing 1 (SVEP-1); [151] and Von Willebrand factor (VWF) [152] are higher than in healthy ones. Furthermore, these markers showed good correlation with hepatitis B [153].

Although EV-encapsulated HBV particles are protected from direct recognition by immune cells, the EVs themselves are recognized by immunocompetent cells and initiate immune response. For example, EVs taken up by macrophages have been shown to significantly upregulate programmed death ligand-1 (PD-L1), which in turn downregulates the CD69 inhibitor of T cell activation [153]. In hepatitis B, healthy liver cells produce IFN-α, an antiviral protein, and transport it to infected cells, while macrophage EVs contain IFN-α-associated miRNA and deliver it to infected cells [154]. The RIG-1 virus sensor can be activated by EV-HBV in infected cells [154], and this leads to induction of genes that belong to INF family III. RIG proteins compete with viral polymerase to pregenomic viral DNA and, as a result, they block DNA replication [155].

#### 4.2.3. Hepatitis C

Hepatitis C is a blood borne virus that causes inflammation in the liver and often leads to terminal liver disease (WHO, 2022) [156]. EVs play an important role in carrying and transmitting hepatitis C virus (HCV), even entire viral particles [157]. As it was mentioned in the cases of other hepatitis viruses, EV encapsulation can be advantageous for the virus. It hides them from the immune system (i), increases the tropism of the virus (ii) and allows faster entry into the recipient cells through uncanonical receptors. HCV RNA was shown to be stabilized by the binding of argonaute RISC catalytic component 2 (AGO2), heat shock protein 90 (HSP90) and miR-122 in traveling EVs [158]. Moreover, HCV RNAs encapsulated in EVs are protected from neutralizing antibodies [159]. HCV-containing EVs also transport glycoproteins E1 and E2 and miRNAs such as miRNA-19a, miRNA-192 and miRNA-122; moreover, cytokines and additional factors promoting efficient viral replication have also been detected. HCV-EVs carry miRNA-192 that increases TGF-β production [160]. These agents upregulate pro-fibrinogenic genes, thus inducing ECM formation and fibrosis. In addition, EVs released by HCV-infected cells carry anti-immunogenic messages. For example, they diminish host immune response by the induction of RUNXOR lncRNA and RUNX-1 that increase the level of many immunosuppressive molecules, e.g., arginase-1 (ARG1), inducible nitric oxide synthase 2 (iNOS), signal transducer and activator of transcription 3 (STAT3). These EVs carry ROS molecules that can be anti-inflammatory under certain circumstances, e.g., inducing apoptosis in T cells [161,162].

Interestingly, other EVs can stimulate an immune response. For example, HCV particles can be internalized by LSECs through intercellular contact, and this activates INF I and III production. Secreted EVs from these cells are enriched in INF proteins and block virus replication in the recipient cells [163].

**Table 1:** Structural and functional similarities between EVs and viruses.

	EVs	Viruses	Reference
Size	30–1000 nm	30–1000 nm	[9,10,128,129]
Membrane	Enriched in glycosphingolipids and cholesterol		[9,10,130,133]
Nucleic acids	dsDNA, ssDNA, mRNA, miRNA, lncRNA, fragments	dsDNA, ssDNA, dsRNA, ssRNA	[128,129,130]
Formation	Endosomes	Endosomes, cytoplasm, nucleus	[129]
Transport	ESCRTI-II-III, tetraspannins, ceramide	ESCRTI-II-III, tetraspannins	[131,132]
Secretion	Exocytosis, budding, shedding	Exocytosis	[9,10,132,141]
Density	1.13–1.18 g/L	1.16–1.18 g/L	[128,129]
Alternative secretion pathway	Lipid rafts	Lipid rafts, packed into vesicles	[9,10,163]
Uptake	Clathrin-dependent endocytosis, caveolin-dependent pathway, macropinocytosis, phagocytosis, lipid raft-mediated uptake	Mostly clathrin-dependent endocytosis, caveolin-dependent pathway	[12,13,131]
Final effect on the cells	Proviral (increase the pool of infected cells, modulate immune response in favor of the virus, increase virus binding to the host) Antiviral (supply cells with antiviral proteins, TLR ligands, antigens)	Viral	[132,133,134,135,136,147,148,152]

## 5. Conclusions

Data collected over the last decade have become increasingly convincing that EVs participate in the pathomechanism of various liver diseases, such as NAFLD, ALD, DILI, cancer and infections. Although there are still several questions to answer, which requires extensive research in the future, the usefulness of EVs in the diagnosis and therapy of hepatic diseases is becoming increasingly recognized. The liver has a high regenerative capacity, and it can be promoted by stem cell derived EVs. Modulation of EV secretion by different chemicals or direct EV-based approaches are both future possibilities.

One of the main difficulties hindering the therapeutic use of EVs is caused by the specificities of pharmacokinetics. As mentioned in this review, EV clearance depends on the type, content and size of the vesicles. Although most of the circulating EVs are eliminated rather quickly through hepatocytes, the exact amount and rate are unpredictable, and other organs, such as kidney, spleen and other cells (e.g., macrophages) found in different tissues, are also involved in the process. The complex pharmacokinetics of EVs is highly dependent on the current state of the body.

The uptake of EVs by various cells also increases off-target effects. Since EVs are taken up by macrophages, there is a significant immune reaction with activation of pro-inflammatory genes, yet, as natural carriers, they are still less immunogenic than liposomes.

EVs also have great potential as diagnostic markers for certain liver diseases. Although EVs are present in all body fluids, their use has certain methodological limitations, e.g., inadequacy of equipment, samples contaminated with lipids and proteins, insufficiently documented normalization of results, etc. To date, the FDA has not approved any EV-based therapies for clinical use, but many are in phase I-IV trials. There are a number (approx. 100) of EV studies registered on the www.clinicaltrials.org homepage, and most of them are cancer-related or vaccines.

EVs are very promising in the diagnosis of liver diseases. Currently, precise imaging techniques and liver biopsy are the gold standard, but there is an urgent need to standardize diagnostic markers that can be easily measured in blood. Since EVs play a major role in liver diseases and have recently begun to be studied in detail, this might open up new possibilities for diagnostic development.

As discussed in this review, EVs are involved in many processes in the liver, but the exact mechanisms, regulatory pathways and how these vesicles contribute to liver physiology and pathophysiology are not yet fully understood. EV research is an unexploited area of science with great potential for both diagnostic and therapeutic advances.

## Data Availability

Not applicable.
